# Melatonin protects retinal pigment epithelium cells against ferroptosis in AMD via the PI3K/AKT/MDM2/P53 pathway

**DOI:** 10.3389/fphar.2025.1543575

**Published:** 2025-02-27

**Authors:** Ping Wu, Long Zhao, Yong Du, Jing Lu, Yuxia He, Qinxin Shu, Hui Peng, Xing Wang

**Affiliations:** ^1^ Chongqing Key Laboratory for the Prevention and Treatment of Major Blinding Eye Diseases, Chongqing Eye Institute, Chongqing Branch of National Clinical Research Center for Ocular Diseases, The First Affiliated Hospital of Chongqing Medical University, Chongqing, China; ^2^ Department of Ophthalmology, Chongqing Aier Eye Hospital, Chongqing, China

**Keywords:** ferroptosis, melatonin, retinal pigment epithelium, age-related macular degeneration, Aβ1-40

## Abstract

**Introduction:**

Oxidative stress-prompted degeneration of the retinal pigment epithelium (RPE) notably contributes to the onset of age-related macular degeneration (AMD). However, the pathways leading to RPE deterioration and possible preventative strategies are not yet completely comprehended.

**Methods:**

Ferroptosis was assayed through the evaluation of lipid peroxidation (C11-BODIPY and MDA), reactive oxygen species (ROS), transmission electron microscopy (TEM), iron content measurement, q-PCR, western blotting, and immunofluorescence. To assess the structure and retinal function of RPE in mice, ERG (electroretinography), OCT (optical coherence tomography), and H&E (hematoxylin and eosin) staining were employed. Network pharmacology methods were utilized to elucidate the potential mechanisms underlying melatonin's protective effects against ferroptosis in RPE cells in AMD. Genetic engineering techniques were applied to investigate the regulatory relationships among phosphatidylinositol 3-kinase (PI3K), protein kinase-B (AKT), murine double minute-2 (MDM2), protein 53 (P53), and solute carrier family 7 member 11 (SLC7A11). In vitro knockdown experiments of MDM2 were conducted to explore its regulatory role in ferroptosis within RPE cells.

**Results:**

Aβ1-40 can trigger ferroptosis in RPE cells. Melatonin can inhibit the oxidative stress and ferroptosis induced by Aβ1-40 in RPE cells. Melatonin exhibits a protective effect on Aβ1-40-induced AMD, significantly improving the structure of the mouse retina and RPE layer, and facilitating the restoration of visual function. Network pharmacology methods revealed that the potential targets of melatonin in AMD are closely related to ferroptosis, and indicated that the predominant pathways are significantly associated with the PI3K/AKT/MDM2/P53 signaling pathway. Knocking down the specific expression of MDM2 can significantly weaken the inhibitory effect of melatonin on oxidative stress and ferroptosis.

**Discussion:**

Melatonin can suppress cell death by ferroptosis in RPE via the PI3K/AKT/MDM2/P53 pathway, thereby preventing and decelerating the progression of AMD.

## 1 Introduction

Retinal pigment epithelium (RPE) cells, positioned as a polarized monolayer at the juncture of the outer retina and the choroid, play a crucial role in preserving the structural and functional stability of the environment it inhabits, particularly on both the apical (photoreceptors) and basal (Bruch’s membrane and choroidal blood supply) sides (Hansman et al., 2024; Paraoan et al., 2020). RPE cells, characterized by their heightened metabolic requirements, dense mitochondrial concentration, high levels of reactive oxygen species, and abundant blood flow in the macula, are situated in a highly oxidative environment. Subsequently, oxidative stress-mediated damage to these cells is a key factor in the development of Age-related macular degeneration (AMD) (Dieguez et al., 2019). AMD (Age-related Macular Degeneration) is a common ocular disorder, regarded as a significant factor contributing to central vision impairment in people aged 50 and above (Deng et al., 2022; Guymer and Campbell, 2023). Key risk factors for AMD encompass aging (above 50 years), environmental impacts, and genetic susceptibility (Basyal et al., 2024). Among the environmental impacts, smoking, exposure to blue light, and consumption of a high-fat diet stand out (Basyal et al., 2024; Cougnard-Gregoire et al., 2023). Moreover, people with a familial history of AMD or who carry genetic markers for AMD susceptibility are also at a heightened risk (Bhumika and Bora, 2024). These factors can interact, damaging the macula and initiating an inflammatory feedback loop, which may eventually result in central vision impairment or loss (Kaarniranta et al., 2020). Clinically, progress has been made in delaying wet AMD by inhibiting the growth of new blood vessels, but about 30% of wet AMD patients still experience no improvement in vision (Deng et al., 2022). Numerous risk factors are associated with dry AMD, yet an effective therapeutic approach has not been established to date (Cabral de Guimaraes et al., 2022; Kim and Lad, 2021). Therefore, exploring therapeutic drugs, targets, and mechanisms of action for early AMD, especially dry AMD, is of great significance.

Ferroptosis is a type of regulated cell demise requiring iron, primarily characterized by the overabundance of iron ions, reactive oxygen species (ROS) derived from metabolic byproducts, and phospholipids with polyunsaturated fatty acid chains (PUFA-PL). This results in the peroxidation of lipids in certain cell and cytoplasmic membranes, thereby inducing ferroptosis (Jiang et al., 2021). Ferroptosis significantly affects the course of diseases, including malignant growths and degenerative neurological afflictions (Tang D. et al., 2021), but its mechanism of action in AMD remains to be elucidated. Iron is vital for the visual phototransduction in the retina. Yet, an overload of iron, along with a decline in antioxidant defenses, increases the vulnerability of aged retinas to cell damage triggered by oxidative stress (Ugarte et al., 2013). Potential mechanisms underlying AMD encompass oxidative stress-induced cell death in retinal pigment epithelium (RPE) cells, leading to subsequent death of photoreceptor cells in the retina (Kaufmann and Han, 2024). Grasping the mechanisms responsible for the disturbance of iron and redox balance in RPE cells is essential for elucidating how ferroptosis contributes to the development of AMD (Zhao et al., 2021). Ferroptosis is significantly involved in the development of AMD, as indicated by the alleviation of RPE cell loss, photoreceptor death, and retinal dysfunction by Ferrostatin-1, which is triggered by sodium iodate (Yang et al., 2022).

Melatonin (MEL), an endocrine hormone with neurologic functions, is predominantly synthesized and discharged by the pineal gland following a circadian rhythm (Boutin et al., 2024). In addition to regulating biological rhythms and other physiological functions, melatonin can also regulate blood pressure, act as an antioxidant, protect against apoptosis, safeguard cells from oxidative stress damage, and has immunomodulatory and anti-tumor effects across various biological fields (Galano and Reiter, 2018). Melatonin exerts influence on the mitochondria and modulates retinal oxidative stress, inflammation, and apoptosis. It has been demonstrated to safeguard photoreceptors and the RPE from photic injury, implying that ferroptosis might significantly contribute to the etiology of AMD (Mehrzadi et al., 2020). These studies suggest that melatonin has the potential to prevent and treat AMD. The PI3K/AKT signaling pathway regulates various fundamental cellular functions, including cell growth, proliferation, and survival (Xie et al., 2019). Relevant studies have demonstrated that melatonin stimulates the PI3K/AKT pathway via the MT1 and MT2 receptor pathways (Wang et al., 2019). Phosphorylated AKT can stimulate the ubiquitin ligase MDM2 of P53 through phosphorylative modification, thereby inhibiting the expression of P53 (Acauan et al., 2015). Research suggests that an increase in P53 expression leads to a decrease in SLC7A11 and GPX4 expression, which in turn speeds up ferroptosis in cells (Kang et al., 2019). Therefore, we speculate that melatonin could inhibit ferroptosis in RPE through related mechanisms, thus serving a therapeutic role in AMD. However, the molecular mechanism by which melatonin ameliorates AMD by mitigating specific death of RPE cells remains unclear and warrants further investigation.

Our previous research has used Aβ1-40 to construct a dry AMD model (Chen J. et al., 2022). This study confirmed that ferroptosis occurred in RPE cells induced by Aβ1-40. We further investigated the neuroprotective role of melatonin in ferroptosis and its pathways of action, which holds substantial importance for a deeper comprehension of melatonin’s function in dry AMD.

## 2 Materials and methods

### 2.1 Obtaining molecular frameworks and therapeutic targets of melatonin

The PubChem is an open-access repository of chemical information (Kim, 2016). Informations related to melatonin, such as CID, CAS number, SMILESE format, 2D and 3D structure, etc. can be obtained through PubChem database, which is beneficial for the identification of potential melatonin targets. The corresponding information of melatonin was input into TCMSP database (Yee et al., 2024), Swiss Target Prediction database (Fu et al., 2023), drugbank database (Reisdorf et al., 2017), pharmmapper database (Huang et al., 2018), etc., to find all the melatonin target proteins of melatonin as far as possible. The Uniprot database (Bowler-Barnett et al., 2023) facilitated the conversion of melatonin’s target proteins into their equivalent canonical gene nomenclature, specified for “Human” species, which were then uploaded to the STRING database (Crosara et al., 2018) for the elimination of disconnected targets, resulting in a network diagram of melatonin’s target genes.

### 2.2 Identification of potential therapeutic targets for melatonin in AMD

To further investigate the medicinal action of melatonin in the context of AMD, our study draws on research ideas similar to those reported in other network pharmacology. The Target of AMD disease was screened by GeneCards database, Swiss Target Prediction database and pharmmapper database, and The official gene nomenclature of the corresponding targets was acquired. Wayne analysis was conducted on the WeisenXin analytical tool (Tang et al., 2023) to map the targets of melatonin and AMD, identifying therapeutic candidates for age-related macular degeneration and organizing the potential targets of melatonin in AMD treatment.

### 2.3 Analyzing the network of intersecting genetic elements

Incorporating the intersecting targets into the STRING database, we assembled the protein-protein interaction (PPI) network model and imported it into Cytoscape 3.9.1 software. Employing the CytoNCA plugin, we analyzed the topological parameters of each node within the network to construct the core target selection network. Using a network analysis approach, we analyzed the degree information of PPI network nodes and selected the top 20 genes (with a degree of at least 13) as key targets. FerrDb database (Zhou et al., 2023) integrates data from 3,429 iron-related literature in PubMed, offering information on 232 iron-related diseases, 264 iron-driven genes, 238 iron-inhibitory genes, 298 iron-inducers, and 249 iron-inhibitors. As of the current version, these data continue to be updated. We utilized the FerrDb database to understand the relationship between the top 20° targets and ferroptosis.

### 2.4 Analysis of KEGG pathways and molecular docking of potential targets of melatonin in AMD

Integrating the intersecting target genes into the Metascape platform (Zhou et al., 2019) facilitated GO and KEGG enrichment analyses, which were visualized using an OMICS analytics platform. Melatonin’s structure was illustrated with ChemDraw software, and its PDB file was constructed in Discovery Studio 2019, refined in AutoDockTools 1.5.6, and archived in PDBQT format. The human PI3K protein’s PDB file was retrieved from the Protein Data Bank (PDB), processed in AutoDockTools 1.5.6, and saved in PDBQT format. AutoDock Vina 1.1.2 was employed for the preprocessing and optimization of the receptor protein and ligand molecules, with the docking box delineated around the PI3K’s active site, and the semi-flexible docking approach was executed to acquire the docking results of melatonin with the human PI3K protein.

### 2.5 Cell culture

The ARPE-19 cell line (ATCC, USA) was cultured in DMEM/F12 medium (1:1) (Procell Life Science & Technology, Wuhan, China), and incubated in a 37°C incubator (Thermo Fisher, USA) with 5% CO2. The culture medium was replaced 1 day before the experiment, with 1 × 10 ^ 4 cells per well for 96-well plates, 4 × 10 ^ 5 cells per well for 6-well plates, 2 × 10 ^ 4 cells per well for 24-well plates, 5 × 10 ^ 5 cells per well for 6 cm dishes. Prior to further experimentation, cells were pretreated with melatonin at concentrations ranging from 0 to 200 μM (inclusive of 10 μM, 50 μM, and 100 μM) for a duration of 24 h. Subsequently, the cells were subjected to either a 48-h treatment with Aβ1-40 dissolved in DMSO at a concentration of 10 μM or a 24-h treatment with erastin dissolved in DMSO at a concentration of 5 μM.

### 2.6 Assessments of cellular viability *in vitro*

Cells were seeded into 96-well plates at a density of 1 × 10 ^ 4 cells per well with five replicate wells per group using the CCK-8 assay kit (MeiLun Biotech, Dalian, China). Following an initial 24-h incubation, the cells were primed with several concentrations of Melatonin for 24 h before being activated with Aβ1-40 at 10 μM for 48 h. CCK-8 assay solution (10 μL) was applied to each well, and after incubating for intervals between 0.5 and 4 h, the absorbance of the samples was assessed at 450 nm with a microplate photometer. The relative cell viability was calculated in comparison to the blank control group.

### 2.7 siRNA transfection

MDM2 siRNA (GenePharma, Shanghai, China) was utilized to suppress the expression of MDM2.The Si-MDM2 sequence is: 5′- CAG​GCA​AAT​GTG​CAA​TAC​CAA​CA -3′. Cells were inoculated into 6-well plates at a rate of 4 × 10 ^ 5 cells per well, and they were cultivated to achieve 60%–70% confluence before the transfection procedure. The transfection was carried out using gp-Transfection-Mate (GenePharma, Shanghai, China) with MDM2 siRNA or NC siRNA at a concluding level of 20 nM. After 8 h of MDM2 siRNA transfection in ARPE-19 cells, the medium was replaced with complete growth medium, and the cells were incubated overnight before proceeding to the next steps of the experiment.

### 2.8 Transmission electron microscope

Following various interventions on ARPE-19 cells, the cells underwent overnight fixation in 2.5% glutaraldehyde phosphate at 4°C, followed by postfixation in 2% osmium tetroxide buffer, dehydration, and embedding in Epon812 resin (Merck). Slices of 70 nm in thickness were prepared and then stained with uranyl acetate and lead citrate, and subsequently analyzed under a transmission electron microscope (HITACHI, HT7800, Japan).

### 2.9 Iron content analysis and detection of LPO (lipid peroxidation)

ARPE-10 cells were seeded in six-well plates and incubated at 37°C in a 5% CO2 incubator overnight. After treatment with various interventions, intracellular Fe2+ and LOS were visualized using fluorescence imaging. FerroOrange (Dojindo Laboratories, Kumamoto, Japan) at 1 μM was added to the cells and incubated for 30 min before observation with a Leica inverted fluorescence microscope. The C11-BODIPY 581/591(Thermo Fisher Scientific, USA) at 10 μM was introduced to the cells, incubated for 30 min, and then observed using the same microscope. Fluorescence imaging was performed at Texas Red (590 nm) and FITC (510 nm) channels, and the fluorescence intensity ratio at these wavelengths was calculated to assess the level of lipid peroxidation.

### 2.10 MDA measurement

Post various interventions on ARPE-19 cells, cells were lysed for supernatant collection. Assessment of protein levels was conducted with the aid of a BCA protein quantification kit. A mixture of 0.1 mL supernatant and 0.2 mL TBA was heated at 100°C for 15 min, cooled to room temperature, and centrifuged at room temperature with 1000 g for 10 min. A 200 μL portion of the supernatant was pipetted into a 96-well plate, and the absorbance at 532 nm was recorded with a microplate reader to assess MDA concentrations.

### 2.11 GSH measurement

Adequate numbers of cells (≥10^6^) were collected for each sample, subjected to two to three freeze-thaw cycles between freezing in liquid nitrogen and thawing in a 37°C water bath, then centrifuged to obtain the supernatant which was kept on ice. Protein concentration in the supernatant was evaluated. A corresponding volume of supernatant was blended with GSH reagent in the prescribed proportion, incubated at room temperature for 2 min, and thereafter, the absorbance was quantified using a plate reader to compute the GSH content.

### 2.12 ROS measurement

Post a 48-h period of plating and drug treatment, 1 mL of DCTH-DA at 10 μM per well was added and kept in the dark at 37°C for 20 min. The cells were rinsed three times with PBS before being observed under a Leica inverted fluorescence microscope from Germany.

### 2.13 Western blot assay

Extraction of proteins from ARPE-19 cells and mouse RPE-choroid complexes was facilitated by RIPA buffer, enhanced with protease inhibitor formulations, and protein concentrations were determined with a BCA protein assay kit (Meilunbio, Dalian, China). A fivefold concentrated protein loading buffer was added at a 4:1 dilution, followed by heating for 5–10 min to denature proteins. Equivalent quantities of protein (20 μg for cellular samples and 40 μg for ocular samples) were resolved using a 4%–20% gradient SDS-PAGE gel and then transferred to a 0.45 μm PVDF membrane. After blocking with 5% non-fat milk at ambient temperature for 2 h, the membrane was subsequently incubated with the primary antibodies (Anti-TFR, Anti-ACSL4, Anti-NRF2, Anti-SLC7A11, Anti-Gpx4, Anti-FTH1, Anti-β-actin, Anti-P-PI3K, Anti-PI3K, Anti-P-AKT, Anti-AKT, Anti-P-MDM2, Anti-MDM2, Anti-P53) with overnight incubation at 4°C. The ensuing day, the protein bands were treated with HRP-linked secondary antibodies, goat anti-rabbit or anti-mouse IgG (H&L), diluted to 1:10,000, for a duration of 1–2 h at ambient temperature. The bands were identified using an ECL luminescent reagent and imaged with a gel imaging system, with β-Actin as a reference control.

### 2.14 RNA extraction and q-PCR

Total RNA was extracted from post-intervention ARPE-19 cells or murine retina-RPE-choroid complex using TRIzol reagent and subsequently reverse transcribed into cDNA. Quantitative real-time PCR was conducted on the ThermoFisher 7,500 Real-Time PCR system, Singapore, to ascertain gene transcription levels. The details of the primers used in this research are presented in Table 1.

**TABLE 1:** Sequences of PCR primers.

Gene	Forward primer	Reverse primer
ACSL-4	GCT​ACT​TGC​CTT​TGG​CTC​A	GGT​CAG​AGA​GTG​TAA​GCG​GA
Gpx4	GAG​GCA​AGA​CCG​AAG​TAA​ACT​AC	CCG​AAC​TGG​TTA​CAC​GGG​AA
TFRC	CTG​AAC​CAA​TAC​AGA​GCA​GAC​A	GGA​AGT​AGC​ACG​GAA​GAA​GTC
SLC7A11	TCT​CCA​AAG​GAG​GTT​ACC​TGC	AGA​CTC​CCC​TCA​GTA​AAG​TGA​C
GSH	GGG​AGC​CTC​TTG​CAG​GAT​AAA	GAA​TGG​GGC​ATA​GCT​CAC​CAC
Β-actin	CAT​CCG​TAA​AGA​CCT​CTA​TGC​CAA​C	ATG​GAG​CCA​CCG​ATC​CAC​A

Table 1 RT-PCR primer sequences are as follows. The abbreviation list includes: ACSL-4 for acyl-CoA synthetase long chain family member 4; GPX4 for glutathione peroxidase 4; TFRC for transferrin receptor; SLC7A11 for solute carrier family 7 member 11; and GSH for glutathione synthetase.

### 2.15 Immunocytochemistry (ICC) analysis

The ARPE-19 cell line was cultured in 24-well plates that featured glass coverslips at the base. Following the experimental treatment, the cells were rinsed using PBS and subjected to 4% paraformaldehyde fixation for 15 min. Cell permeabilization was facilitated using a 0.5% concentration of Triton X-100 detergent in PBS for 5 min, followed by incubation with a 10% goat serum solution at room temperature for 1 h to avoid non-specific antibody interactions. Coverslips from 24-well plates were detached, and post-serum removal, cells underwent primary antibody incubation at 4°C overnight or 37°C for 2 h. Following specimen washing, they were incubated with a FITC-labeled secondary antibody in darkness at room temperature for 1 h. Subsequently, cell nuclei were subjected to DAPI staining for 5 min. An antiquenching mounting solution was applied to inhibit fluorescence decay, followed by microscopic observation and documentation of the cells.

### 2.16 Haematoxylin and eosin staining (HE)

Ocular tissues from treated mice were excised and preserved in 4% neutral-buffered formalin for 24 h, then processed through a graded ethanol series and encapsulated in paraffin. Paraffin-embedded blocks were secured to a microtome, from which thin sections (5–8 μm) were produced and transferred onto glass slides. Paraffin-embedded sections were cleared of wax, followed by H&E staining, and subsequently mounted and resin-fixed. Observation and photography of the tissue structure were conducted using an inverted microscope.

### 2.17 Optical coherence tomography (OCT)

After anesthesia intervention on the mice, tropicamide was administered to dilate their pupils, followed by fundus scanning using SS-OCT to obtain clear OCT images of the fundus.

### 2.18 Electroretinography (ERG)

The mice underwent an intervention and were then subjected to a 12-h dark adaptation period before the next experiment. While the mice were anesthetized, pre-treatment was administered, and mydriasis was induced using red light. The reference and ground electrodes were attached to the forehead and tail, respectively, while the electrode on the cornea was carefully positioned on the test eye. The examination was conducted using the PuREC system with Version 3.0.0 (MAYO CORPORATION, Japan). After the baseline stabilized, four different light stimulus intensities (0.9, 1.8, 3.0, 3.9 log cd·sec/m^2^) were used for detection, and the recorded waveforms and numerical data were saved. All ERGs (electroretinograms) were conducted at the same time of day.

### 2.19 Animal

Compliance with the ARVO Statement was maintained throughout all experiments, and the institutional animal ethics committee at Chongqing Medical University (Ethics code: Chongqing, China, IACUC-CQMU-2023–0355) endorsed all project-affiliated animal procedures. Male C57BL/6 strain mice, weighing 20–25 g and 6–8 weeks of age, were obtained from the Experimental Animal Research Center of Chongqing Medical University. Subsequently, we euthanized the mice by cervical dislocation under anesthesia.

### 2.20 Animal treatment

**FIGURE 1:**
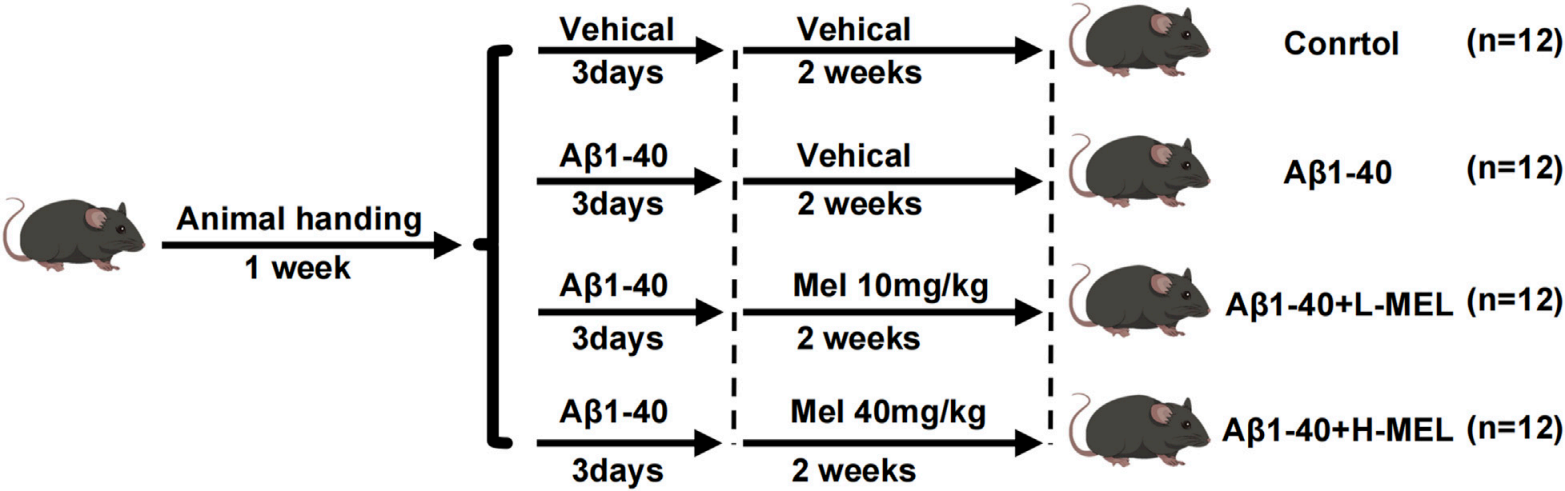
Timeline of experimental design *in vivo*. Mice were allocated across four groups: Control, Aβ1-40 only, low-dose melatonin (10 mg/kg) with Aβ1-40 (Aβ1-40 + L-Mel), and high-dose melatonin (40 mg/kg) with Aβ1-40 (Aβ1-40 + H-Mel).

In in vivo experiments, mice were anesthetized with 1.5% pentobarbital sodium at 5 μL/g via intraperitoneal injection. A microsyringe was used under a microscope to inject Aβ1-40 (2 μL of a 350 μM solution) into vitreous cavity of mice in the intervention group, while the control group eyes were injected with 1 × PBS (2 μL) as a control. After 3 days, each mouse received daily intraperitoneal injection of either melatonin at 10 mg/kg, melatonin at 40 mg/kg, or 0.09% saline. After a 2-week pretreatment period, the mice were utilized for subsequent experiments. After SS-OCT imaging for fundus examination in the anesthetized mice, the mice were euthanized, their eyes were enucleated, and either subjected to H&E staining or prepared for separation of the retina-RPE-choroid complex for q-PCR and WB experiments. The experimental timeline is depicted in Figure 1.

### 2.21 Materials

Melatonin, Erastin, and Fer-1 were acquired from MedChemExpress (New Jersey, USA). The CCK-8 assay kit was sourced from Meilun Biotech of China. The Malondialdehyde (MDA) assay kit, GSSG/GSH quantification kit, and ROS kit were acquired from BIOPRIMACY of China. C11-BODIPY(581/591) was purchased from Thermo Scientific (Waltham, MA, USA). Aβ1-40 was obtained from apeptides of China. FerroOrange was acquired from Dojindo (Kumamoto, Kyushu, Japan). ARPE-19 cells were sourced from ATCC(Maryland, USA). DMEM/F12 medium, penicillin/streptomycin, and fetal bovine serum (FBS) were obtained from Procell Life Science & Technology (Wuhan, China). Si-MDM2 was sourced from GenePharma (Shanghai, China). All protein antibodies were obtained from Affinity (China Co., Ltd., China). All q-PCR primers were sourced from agbio (China Co., Ltd., China).

### 2.22 Statistical analysis

All experiments were conducted at least three times, and the results are reported as the mean ± standard error of the mean (SEM ± SD). For normally distributed data, statistical comparisons among multiple groups were conducted using one-way ANOVA with Bonferroni correction. Unpaired t-tests or ordinary two-way ANOVA were used for comparisons between two groups. GraphPad Prism 9.0 software was employed for all statistical analyses. Statistical significance was set at a P-value <0.05, denoted as *P < 0.05, **P < 0.01, ***P < 0.001, and ****P < 0.0001; ns indicates non-significance.

## 3 Results

### 3.1 Ferroptosis is the primary pathological process of Aβ1-40-induced RPE cell degeneration

**FIGURE 2:**
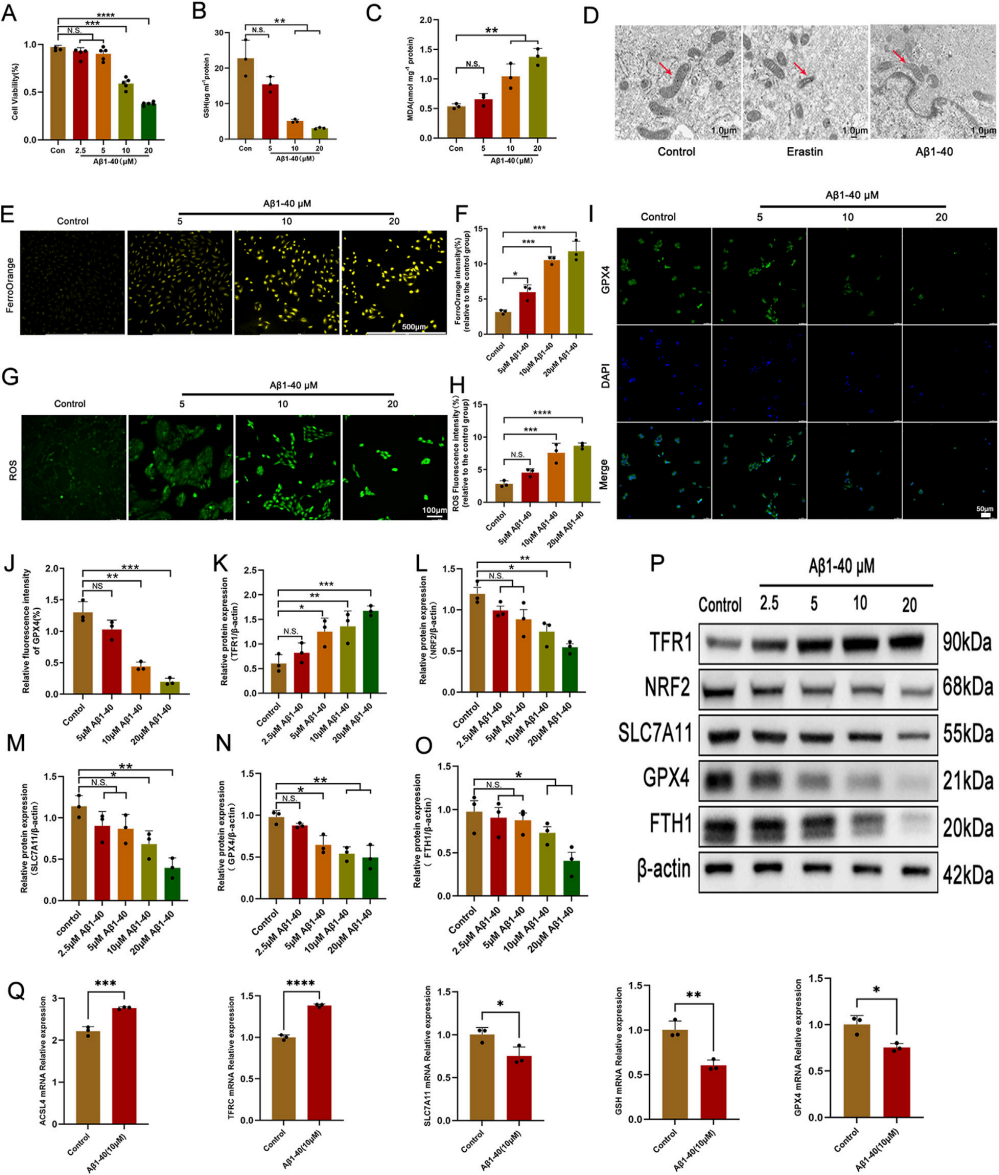
Ferroptosis is the primary pathological process of Aβ1-40-induced RPE cell degeneration **(A)** The ARPE-19 cell was exposed to varying concentrations of Aβ1-40 for 48 h, and a CCK8 assay was utilized to assess cell viability. Following the exposure of ARPE-19 cells to various concentrations of Aβ1-40 for 48 h, the levels of GSH **(B)** and MDA **(C)** were determined. **(D)** TEM imaging of the mitochondrial ultrastructure in ARPE-19 cells after treatment with Erastin at 5 μM and Aβ1-40 at 10 μM for 48 h is shown (red arrows indicate mitochondria, scale bar: 1.0 μm). **(E)** FerroOrange staining (scale bar: 500 μm) was used to assess intracellular ferrous ion levels, with results quantitatively analyzed using **(F)** ImageJ. ROS levels were detected with **(G)** DCFH-DA staining (scale bar: 100 μm) and quantitatively analyzed with **(H)** ImageJ. **(I)** Immunofluorescence (scale bar: 50 μm) was employed to evaluate GPX4 protein expression levels, with quantitative analysis performed using **(J)** ImageJ. **(K–O)** Semi-quantitative **(P)** Western blot analysis was conducted to detect protein expression levels of TFR1, NRF2, SLC7A11, GPX4, and FTH1 in cells. **(Q)** mRNA expression levels of ACSL4, TFRC, SLC7A11, GSH, and GPX4 were examined via qPCR analysis. n = 3 (except n = 5 in CCK8), mean ± SD. one-way ANOVA with Bonferroni correction. *p < 0.05, **p < 0.01, ***p < 0.001, and ****p < 0.0001.

Amyloid beta (Aβ) plays a crucial role in the composition of drusen and can induce damage to RPE cells by activating inflammatory responses (Do et al., 2019; Jo et al., 2020). Consequently, it affects the onset and progression of AMD, suggesting that Aβ may be one of the key factors involved in early-stage AMD (Chen J. et al., 2021). Aβ has been utilized to provoke inflammatory injury in RPE cells and replicate the pathological alterations found in AMD eye conditions (!!! INVALID CITATION; Liu et al., 2015). We applied the CCK-8 assay to determine the viability of ARPE-19 cells subsequent to exposure to Aβ1-40. The CCK-8 assay results indicated that, at concentrations of 10 and 20 μmol/L, Aβ1-40 progressively inhibited the viability of ARPE-19 cells (Figure 2A). Considering that 10 μmol/L was the threshold to ensure 50% cell survival, we selected a concentration of 10 μmol/L Aβ1-40 for subsequent experiments. We used GSH and MDA kits to detect the expression of GSH and MDA in ARPE-19 cells. As the concentration of Aβ1-40 increased, the expression of GSH gradually decreased (Figure 2B), while the expression of MDA gradually increased (Figure 2C). we detected that the 48-h administration of Erastin or Aβ1-40 to ARPE-19 cells resulted in heightened mitochondrial membrane density and reduced mitochondrial volume via TEM, signified by red arrows (Figure 2D). Using TEM, we observed that following 48-h treatment of ARPE-19 cells with Erastin or Aβ1-40, there was an increase in mitochondrial membrane density and a decrease in mitochondrial volume (indicated by red arrows) (Figure 2D). Upon staining cells with FerroOrange (for ferrous ion detection), we discovered that cells treated with Aβ1-40 exhibited excessive accumulation of ferrous ions, with a dose-dependent increase in orange fluorescence intensity (Figures 2E, F). By staining cells with DCTH-DA fluorescent probe to detect intracellular ROS expression, we found that green fluorescence intensity increased as the concentration of Aβ1-40 increased (Figures 2G, H). The immunofluorescence results (Figures 2I, J) further suggested that the expression of GPX4 in cells decreased in a concentration-dependent manner following induction by Aβ1-40. We utilized WB analysis to assess the expression of ferroptosis-related proteins in cells and observed that TFR1 expression levels rose in a dose-responsive way, whereas NRF2, SLC7A11, GPX4, and FTH1 expression levels fell in a dose-responsive manner (Figures 2K–P). Furthermore, we employed q-PCR to examine the expression of ferroptosis-related genes in cells and discovered that the expression levels of ACSL4 and TFRC rose in a concentration-dependent manner, whereas the expression levels of SLC7A11, GSH, and GPX4 decreased in a similar concentration-dependent pattern (Figure 2Q).

### 3.2 Melatonin inhibits Aβ1-40-induced ferroptosis in RPE

**FIGURE 3:**
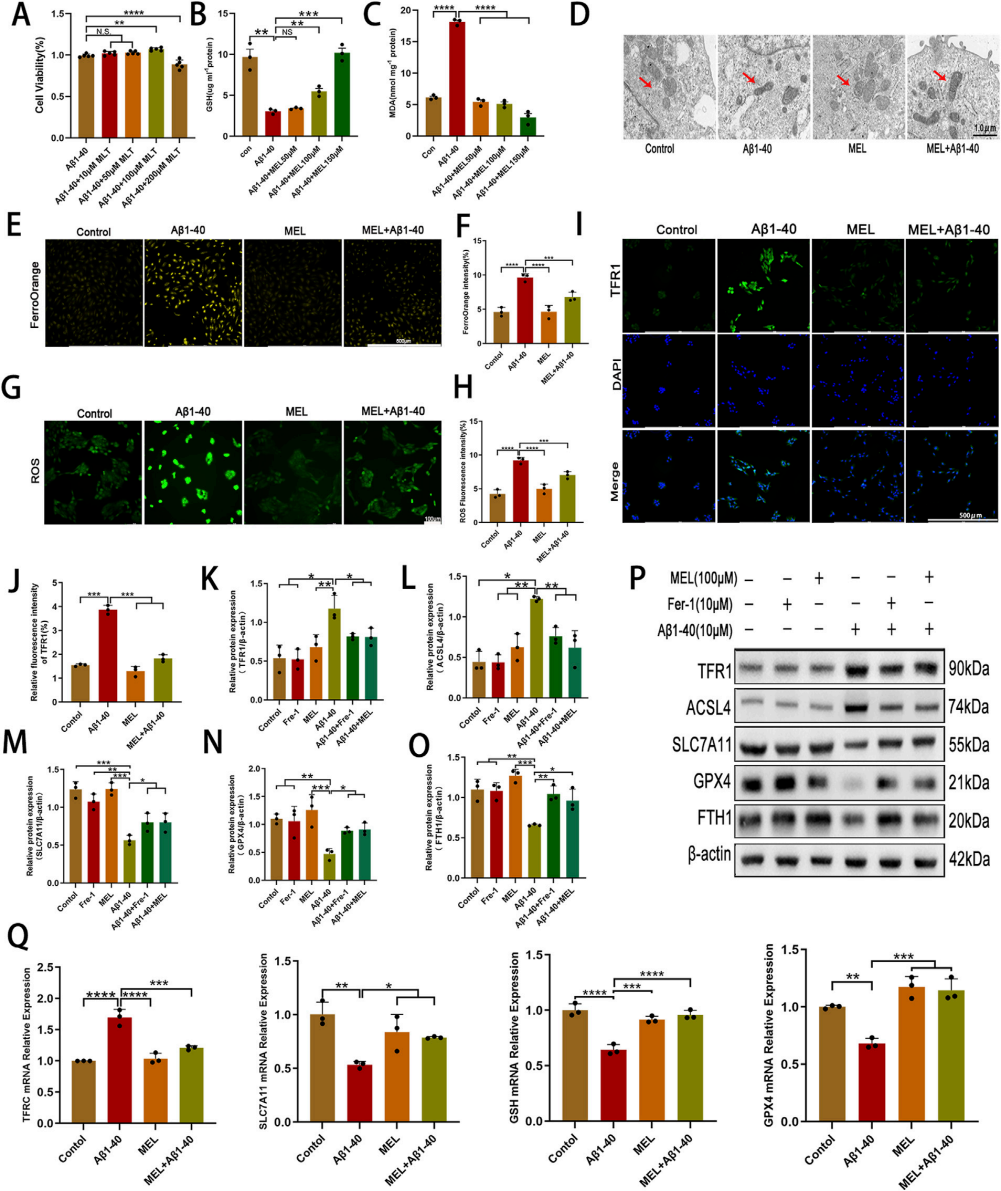
Melatonin inhibits Aβ1-40-induced ferroptosis in RPE cells **(A)** ARPE-19 cells were incubated with diverse concentrations of melatonin for 24 h, then stimulated with 10 μM Aβ1-40 for 48 h, and cell viability was subsequently assessed with CCK-8. After different treatments, **(B)** GSH and **(C)** MDA levels were quantified. After subjecting ARPE-19 cells to various treatment conditions (Aβ1-40, melatonin, and melatonin + Aβ1-40), we examined mitochondrial ultrastructure using **(D)** TEM, with red arrows indicating mitochondria and a scale bar of 1.0 μm. **(E)** FerroOrange stainingwas used to detect ferrous ion levels, with results quantified using **(F)** ImageJ, and **(G)** DCFH-DA stainingwas used to detect reactive oxygen species (ROS), with quantification also performed with **(H)** ImageJ. **(I)** Immunofluorescence analysis was conducted to assess TFR1 protein expression levels, with quantification results obtained using **(J)** ImageJ. Protein expression levels of TFR1, ACSL4, SLC7A11, GPX4, and FTH1 in ARPE-19 cells following various treatment conditions were assessed using **(P)** Western blot analysis and **(K–O)** semi-quantitative detection. **(Q)** Quantitative PCR analysis was conducted to assess the mRNA expression levels of ACSL4, TFRC, SLC7A11, GSH, and GPX4 in ARPE-19 cells after a 32-h period, with some groups pre-treated with melatonin for 24 h, followed by Aβ1-40 treatment for 8 h before testing. n = 3 (except n = 5 in CCK8), mean ± SD, one-way ANOVA with Bonferroni correction, *p < 0.05, **p < 0.01, ***p < 0.001, ****p < 0.0001.

To explore the protective effect of melatonin on Aβ1-40-induced ferroptosis in RPE cells, we initially assessed the impact of a range of melatonin concentrations on the viability of ARPE-19 cells treated with Aβ1-40 using the CCK-8 assay. Results from the CCK-8 assay demonstrated that a melatonin concentration of 100 μmol/L rescued the survival rate of ARPE-19 cells (Figure 3A). We employed GSH and MDA assay kits to evaluate the impact of varying concentrations of melatonin on the levels of GSH and MDA in Aβ1-40-induced ARPE-19 cells. As the concentration of melatonin increased, the levels of GSH gradually increased (Figure 3B), and the levels of MDA gradually decreased (Figure 3C). Using TEM, we observed that ARPE-19 cells treated with both melatonin and Aβ1-40, in contrast to Aβ1-40-only treated cells, displayed higher mitochondrial membrane density and reduced mitochondrial size (mitochondria indicated by red arrows) (Figure 3D). Upon FerroOrange staining for ferrous ion detection, we observed that cells treated with Aβ1-40 accumulated an excess of ferrous ions, whereas those cotreated with melatonin and Aβ1-40 exhibited markedly decreased orange fluorescence (Figures 3E, F). By staining cells with DCTH-DA fluorescent probe to detect intracellular ROS expression, we observed enhanced green fluorescence in ARPE-19 cells treated with Aβ1-40, while the fluorescence was markedly reduced in cells co-treated with melatonin and Aβ1-40 (Figures 3G, H). Results from immunofluorescence assays (Figures 3I, J) demonstrated that TFR1 expression levels were lower in the melatonin + Aβ1-40 group as compared to the Aβ1-40. To further investigate the protective effects of Fer-1, an inhibitor of ferroptosis, and melatonin on the ARPE-19 cell line, Western blot analysis was employed to assess the expression levels of proteins associated with ferroptosis within the cells. We observed that Aβ1-40 treatment increased TFR1 and ACSL4, while reducing SLC7A11, GPX4, and FTH1. However, cotreatment with Fer-1 + Aβ1-40 and melatonin + Aβ1-40 groups restored the expression of these proteins related to ferroptosis (Figures 3K–P). Furthermore, q-PCR was employed to assess the expression levels of genes associated with ferroptosis within cells. We observed that the expression level of TFRC was higher in the Aβ1-40-exposed group compared to the Control, whereas the expression levels of SLC7A11, GSH, and GPX4 were lower. However, the expression patterns of these genes were restored in the group with melatonin cotreatment with Aβ1-40 (Figure 3Q).

### 3.3 Identification and evaluation of putative therapeutic targets for AMD mediated by melatonin

**FIGURE 4:**
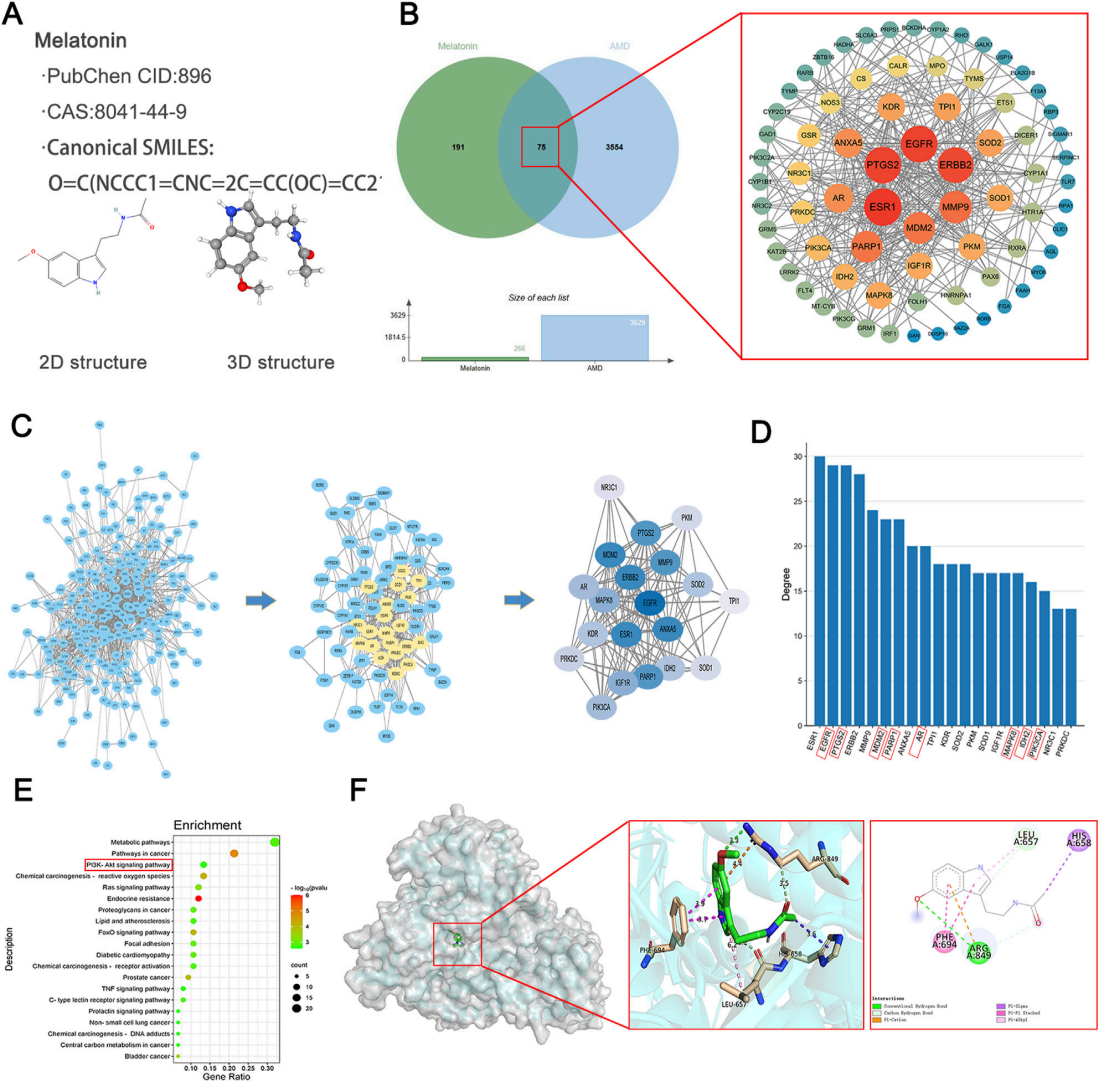
Identification and Evaluation of Putative Therapeutic Targets for AMD Mediated by Melatonin. **(A)** Information and 2D, 3D structures of melatonin. **(B)** Venn diagram illustrating the interaction between melatonin and AMD, along with the construction of a core target PPI (Protein-Protein Interaction) network diagram. **(C)** The comprehensive target set of melatonin, candidate targets of melatonin in AMD (convergence of therapeutic and pathological targets), and the top 20 targets based on degree ranking in the protein-protein interaction (PPI) network assessment for melatonin’s potential targets in AMD. **(D)** The top 20 targets ranked by connectivity values in the protein-protein interaction network analysis for melatonin’s potential targets in AMD, with genes highlighted in red diagonal boxes being related to ferroptosis. **(E)** KEGG pathway enrichment analysis for melatonin’s candidate targets associated with AMD, highlighting red-boxed pathways as potentially influenced by melatonin. **(F)** Molecular docking studies exploring interactions between melatonin and genes implicated in therapeutic pathways.

Two-dimensional and three-dimensional structures of melatonin, along with associated data, were obtained from PubChem (Compound ID 896; CAS number 8041–44-9, etc.) (Figure 4A). By searching through literature and four databases (Drugbank, TCSMP, SwissTargetPrediction, and PharmMapper), a total of 266 melatonin target genes were obtained. Furthermore, a total of 3629 AMD-associated targets were extracted from GeneCards. We entered the selected drug and disease targets into the web-based bioinformatics tool at http://www.bioinformatics.com.cn/and discovered 75 common genes. To comprehend the full spectrum of cellular functional interactions between gene expression and associated proteins, and to elucidate the roles of these proteins in cellular expression, the identified targets were inputted into the STRING database for the purpose of building a model of protein-protein interactions (PPI) network. The resulting network model was loaded into Cytoscape version 3.9.1, employing the CytoNCA extension to analyze the topological parameters of each node within the network. Significance of network targets was assessed considering three key filtering criteria: connectivity, betweenness, and closeness. This led to the construction of a network of core targets to identify key targets (Figure 4B).

By conducting additional network analysis, the connectivity degree of each node in the aforementioned PPI network was calculated and sorted. The top 20 genes with degree values ≥ 13 were identified as key targets. The first figure depicts all targets of melatonin, the second figure illustrates the potential targets of melatonin for AMD (intersection of drug and disease targets), and the third figure presents the top 20 targets ranked by degree values from the PPI network analysis of potential targets (Figure 4C). We sorted the top 20 genes based on their degree values and presented them in a bar chart. By searching the ferroptosis database, we found that most of these targets are related to ferroptosis (with ferroptosis-related genes marked by red diagonal boxes). These include EGFR (Zhan et al., 2023), PTGS2 (Zhou et al., 2021), MDM2 (Chen et al., 2024), PARP1 (Li et al., 2022), AR (Zhang Z. et al., 2023), MAPK8 (Xia et al., 2023), IDH2 (Peng et al., 2024), PIK3CA (Chen H. et al., 2022), and others (Figure 4D).

To achieve a more profound comprehension of the gene functions and their roles in the signaling pathways of potential targets for AMD related to melatonin, we submitted these targets to Metascape for Gene Ontology-based functional enrichment analysis and KEGG pathway enrichment analysis. With the bioinformatics analytical toolkit, we rendered the enrichment analyses visually. The top 20 signaling pathways from the KEGG results, along with their regulating targets, were used to assemble a network depicting ‘target-pathway’ relationships using Cytoscape 3.9.1 software, with potentially relevant signaling pathways highlighted by red boxes (Figure 4E). Further predicting the interaction pattern and binding affinity between melatonin and proteins within these pathways, we performed molecular docking studies with melatonin and PI3K. The findings revealed an affinity energy of −6.8 kcal/mol between the small molecule and the receptor, suggesting a strong binding interaction. Melatonin mainly interacts with residues on the A chain of PI3K, establishing hydrogen bonds with ARG (Figure 4F).

### 3.4 Melatonin inhibits Aβ1-40-induced ferroptosis in ARPE-19 cells through the PI3K/AKT/MDM2/P53 pathway

**FIGURE 5:**
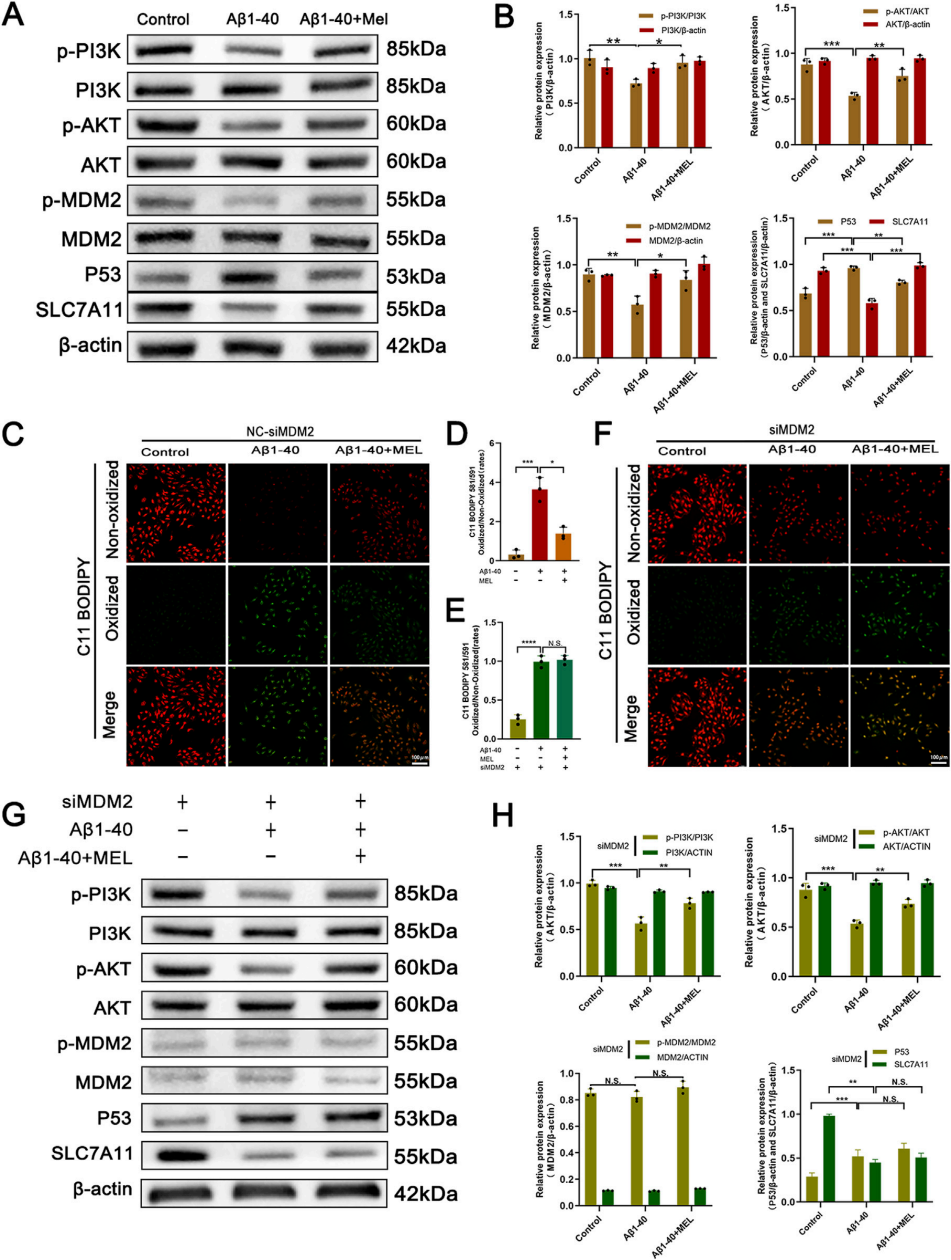
The inhibitory effect of melatonin on ferroptosis in ARPE-19 cells depends on the expression of MDM2 After treating ARPE-19 cells with different interventions (control, Aβ1-40, melatonin + Aβ1-40) for 72 h (cells were pretreated with F12 or melatonin for 24 h, followed by treatment with F12 or Aβ1-40 for 48 h before performing the corresponding assays), the protein expression levels of p-PI3K, PI3K, p-AKT, AKT, p-MDM2, MDM2, P53, and SLC7A11 in the cells were detected by **(A)** Western blot and **(B)** semi-quantitative analysis. Lipid peroxidation in live cells was detected by **(C)** C11-BODIPY staining (scale bar: 100 μm) and quantitatively analyzed using **(D)** ImageJ. After treating ARPE-19 cells with MDM2 knockdown (siMDM2) and the same interventions for 72 h (cells were pretreated with F12 or melatonin for 24 h, followed by treatment with F12 or Aβ1-40 for 48 h before performing the corresponding assays), lipid peroxidation in live cells was detected by **(F)** C11-BODIPY staining (scale bar: 100 μm) and quantitatively analyzed using **(E)** ImageJ. The protein expression levels of p-PI3K, PI3K, p-AKT, AKT, p-MDM2, MDM2, P53, and SLC7A11 in the cells were detected by **(G)** Western blot and **(H)** semi-quantitative analysis. n = 3, mean ± SD, one-way ANOVA with Bonferroni correction, *p < 0.05, **p < 0.01, ***p < 0.001, ****p < 0.0001.

In order to substantiate the process through which melatonin curbs ferroptosis within ARPE-19 cell lines, an initial step involved assessing the protein expression levels subsequent to the application of Aβ1-40 in these cells. Compared to the control group, our analysis indicated a decrease in the concentrations of p-PI3K, p-AKT, and p-MDM2. In contrast, the levels of their non-phosphorylated counterparts (PI3K, AKT, and MDM2) showed negligible changes. The reduced p-MDM2 was unable to exert its inhibitory function on P53, leading to overexpression of P53, which in turn inhibited SLC7A11. However, melatonin reversed this phenomenon (Figures 5A, B). Additionally, we used the C11-BODIPY 581/591 probe to detect intracellular lipid ROS expression and observed this phenomenon using fluorescence imaging. The results indicated that Aβ1-40 induction in ARPE-19 cells enhanced lipid peroxidation (green fluorescence) and weakened the reduced state (red fluorescence), with an increased ratio of the oxidized state to the reduced state. Again, melatonin reversed this phenomenon (Figures 5C, D).

To further investigate the mechanism, we conducted subsequent experiments using ARPE-19 cells transfected with MDM2-siRNA (siMDM2). We employed the C11-BODIPY 581/591 probe to detect intracellular lipid ROS expression, and fluorescence imaging revealed enhanced lipid peroxidation (green fluorescence) and weakened reduction (red fluorescence) in Aβ1-40-induced ARPE-19 cells. However, melatonin was unable to reverse this phenomenon (Figures 5E, F). The Western blot analysis demonstrated a marked reduction in the levels of both phosphorylated MDM2 (p-MDM2) and total MDM2 proteins within the cells following the introduction of MDM2-siRNA, effectively silencing the MDM2 gene. Furthermore, knocking down MDM2 expression had no effect on the expression of upstream signals such as p-PI3K, PI3K, p-AKT, and AKT. Simultaneously, melatonin intervention no longer resulted in the downregulation of the downstream signal P53 and the restoration of SLC7A11 expression.

### 3.5 Experiments demonstrated that melatonin inhibited Aβ1-40-induced ferroptosis in RPE and photoreceptor degeneration *in vivo*

**FIGURE 6:**
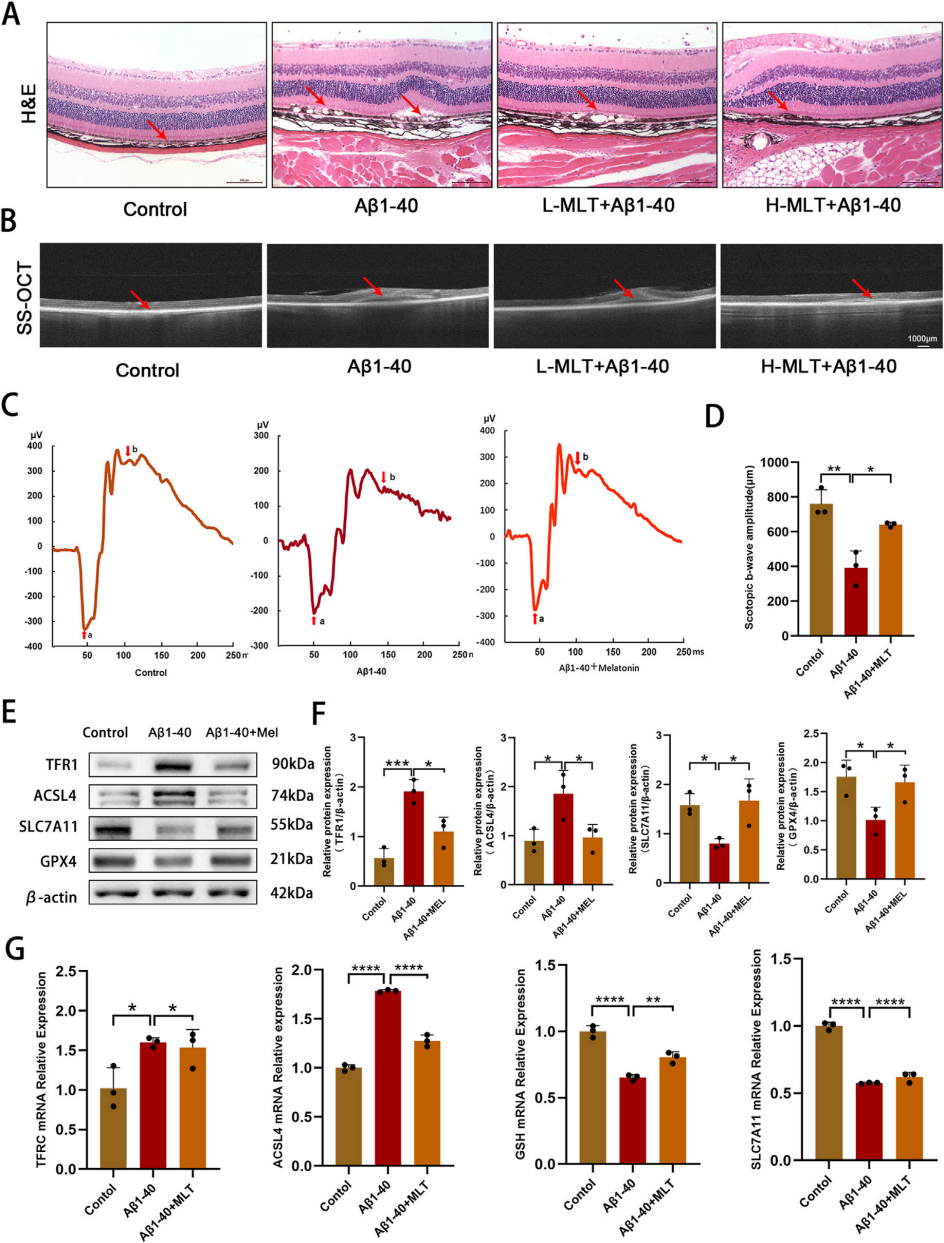
Melatonin have inhibited Aβ1-40-induced ferroptosis in RPE and degeneration of photoreceptors *in vivo*. After intravitreal injection of Aβ1-40 (350 μM/2 mL) for 3 days, mice were treated with different concentrations of melatonin (10 mg/kg, 40 mg/kg) via intraperitoneal injection for 2 weeks. Morphological changes and cell proliferation in the RPE were observed through **(A)** H&E staining (scale bar: 100 μm). Abnormal changes in the RPE and disruption of retinal structure were observed through **(B)** SS-OCT (scale bar: 1,000 μm). The visual function of mice under different interventions (control, Aβ1-40, melatonin + Aβ1-40) was measured using **(C)** full-field ERG after 1 day of dark adaptation, and **(D)** ImageJ was used for quantitative analysis of the amplitude values of the Scotopic B-WAVE. The levels of TFR1, ACSL4, SLC7A11, and GPX4 proteins in the retina-RPE-choroid complex of mice across various treatment groups were identified by employing **(E)** Western blotting and **(F)** semi-quantitative evaluation methods.The transcriptional levels of TFRC, ACSL4, GSH, and SLC7A11 were ascertained utilizing **(G)** q-PCR. n = 3, mean ± SD, one-way ANOVA with Bonferroni correction, *p < 0.05, **p < 0.01, ***p < 0.001, ****p < 0.0001.

In our study, we explored the therapeutic potential of melatonin *in vivo* by administering it daily via intraperitoneal injection to mitigate Aβ1-40-induced ferroptosis in RPE and photoreceptor degeneration in mice. Our results demonstrated that a 2-week melatonin treatment, initiated 3 days post-intraocular Aβ1-40 injection, led to a significant improvement in the morphology and functionality of RPE cells and photoreceptors in the mice. H&E staining revealed disrupted continuity of the RPE layer, loss and swelling of RPE cells (mRPE marked with red arrows) in the retina after Aβ1-40 treatment, while pretreatment with different concentrations of melatonin significantly rescued the RPE (Figure 6A), indicating a satisfactory therapeutic effect of melatonin. SS-OCT examination showed structural disorder and signal weakening of the outer retinal and RPE layers after Aβ1-40 intervention, which was improved by pretreatment with different concentrations of melatonin (Figure 6B). To evaluate the alterations in visual capabilities of mice, we employed full-field ERG to measure the reaction of rod photoreceptors to light exposure in darkness. Compared with normal mice, the ERG curves of mice exposed to Aβ1-40 intervention were significantly reduced, indicating retinal functional damage. Melatonin effectively prevented the decline in magnitudes of the a-wave and b-wave responses in a dark environment after Aβ1-40 induction, demonstrating its improvement of retinal function (Figure 6C). Quantification of the b-wave amplitudes in ERG also suggested melatonin’s improvement of retinal function (Figure 6D). Western blot results showed that compared with normal mice, both TFR1 and ACSL4 were upregulated, while SLC7A11, GPX4, and FTH1 were downregulated in the retina-RPE-choroid complex of mice exposed to Aβ1-40 intervention. However, in mice treated with melatonin, the expression levels of these proteins were restored (Figures 6E, F). Additionally, we validated the impact of melatonin on genes associated with ferroptosis within the retina-RPE-choroid complex tissue using q-PCR. The q-PCR data revealed that in mice subjected to Aβ1-40 treatment, there was an increase in the expression of TFRC and ACSL4, and a decrease in GSH and SLC7A11 in contrast to the control group. However, the mRNA levels of these genes were significantly restored to normal after the introduction of melatonin (Figure 6G).

## 4 Discussion

**FIGURE 7:**
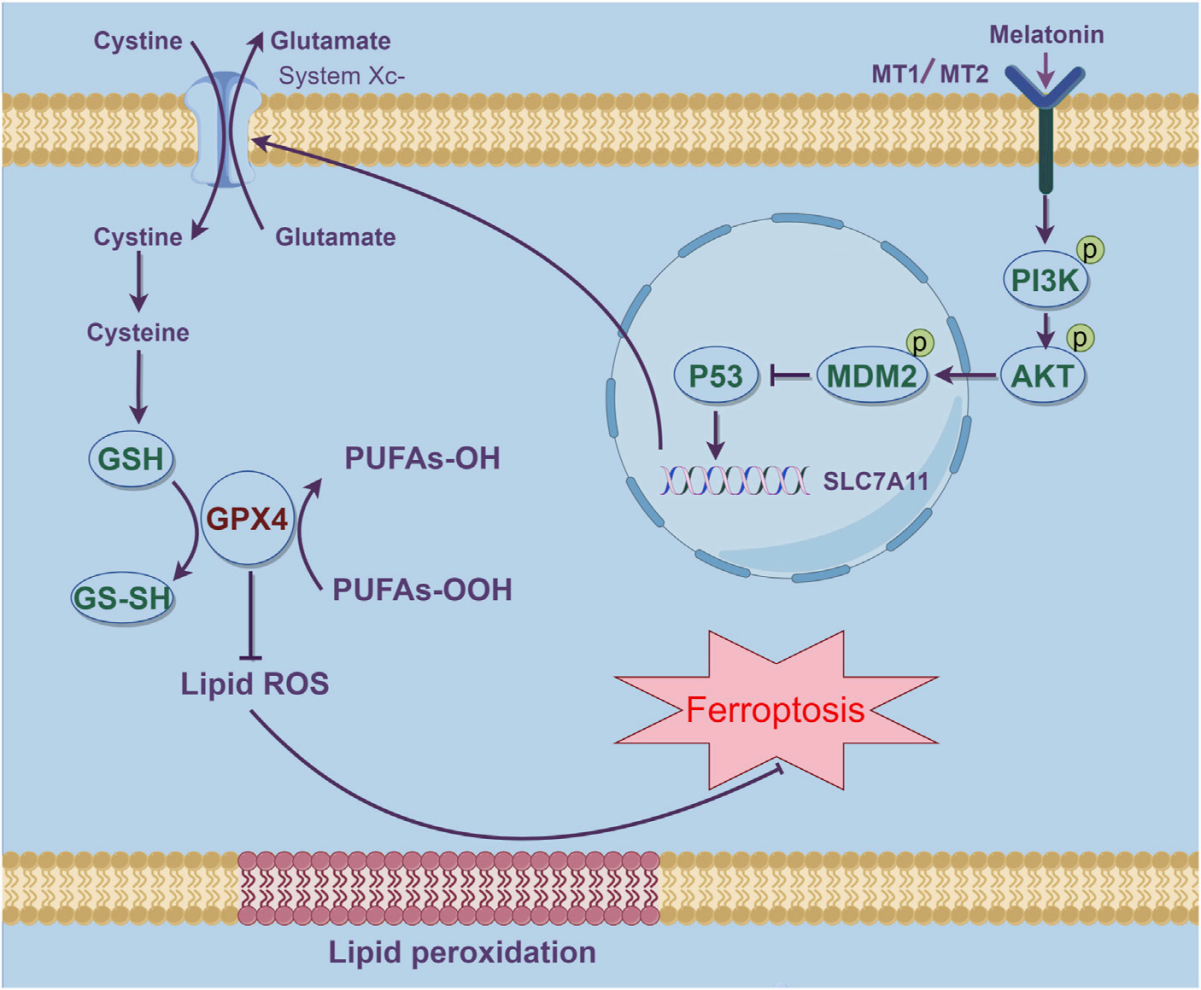
Schematic representation of how melatonin suppresses ferroptosis and ameliorates AMD via the PI3K-AKT-driven MDM2/P53/SLC7A11 pathway. The stimulation of MDM2 results in the inhibition of P53, thereby effectively reducing ferroptosis in AMD.This figure was created using Figdraw.

AMD is a prevalent condition responsible for significant vision loss among the elderly. Its etiology is multifactorial, involving advanced age, genetic predispositions, and environmental influences. The condition progresses as extracellular deposits build up in the retina’s outer layers, culminating in the degeneration of photoreceptors and the consequent loss of central vision (Fleckenstein et al., 2024). Currently, there is no effective treatment for early AMD, particularly the dry AMD. Management focuses on reducing risk factors and using dietary supplements (Schultz et al., 2021). Therefore, the development of new drugs or treatment methods for AMD holds significant importance. In this study, we found that both mouse and cellular models induced by Aβ1-40 exhibited significant oxidative stress and ferroptosis. Melatonin effectively alleviated Aβ1-40-induced ferroptosis through the PI3K/AKT/MDM2/P53 axis (Figure 7), Offering a novel therapeutic strategy for the management of age-related macular degeneration (AMD).

Ferroptosis represents a distinct type of cell demise characterized by iron-mediated phospholipid peroxidation, which is governed by an array of cellular metabolic processes. These include the management of iron, maintenance of redox balance, mitochondrial function, and signaling pathways implicated in various diseases (Jiang et al., 2021; Tang D. et al., 2021). Recent studies indicate that ferroptosis plays a role in the pathogenesis and progression of a variety of diseases, including but not limited to cancer (Chen X. et al., 2021), Parkinson’s disease (Bao et al., 2021), kidney disease (Gupta et al., 2023), and others. Emerging research further supports the notion that ferroptosis contributes to the initiation and advancement of AMD. Research conducted by Urvi Gupta and colleagues has shown that elevated levels of LCN2 (lipocalin-2) in the RPE of dry AMD murine models lead to a decrease in autophagy, triggering the inflammasome and initiating ferroptosis (Gupta et al., 2023). Zhimin Tang and team have identified that modulating HO-1-driven ferroptosis in RPE cells represents a potent approach for retinal preservation, potentially preventing the onset of AMD (Tang Z. et al., 2021). Amyloid beta (Aβ), a key constituent of drusen, is capable of inflicting harm on the RPE cells through the activation of inflammatory pathways, which in turn, can expedite the development of the early stages of AMD (Muraleva et al., 2019). We hypothesize that Amyloid beta can also exacerbate the progression of AMD by inducing ferroptosis in RPE cells. In this study, we found that Aβ1-40 induction in ARPE-19 cells led to increased mitochondrial membrane density and smaller mitochondria (Figure 2), which are important characteristics of ferroptosis (Khatun et al., 2024), and similar findings have been reported in previous literature (Tang Z. et al., 2021). Accumulation of intracellular Fe2+ is one of the typical manifestations of ferroptosis. We detected increased Fe2+ levels in Aβ1-40-induced cells using FerroOrange staining. The increased Fe2+ specifically elevated levels of oxidative stress and lipid peroxidation, including increased expression of MDA and ROS, and decreased expression of GSH which is an important protein that inhibits ferroptosis. We confirmed abnormal expression of ferroptosis-related molecules ACSL4, TFR1, NRF2, SLC7A11, GPX4, and FTH1 through WB, immunofluorescence, and q-PCR. These studies demonstrate that ferroptosis is the primary pathological process by which Aβ1-40-induced oxidative stress causes RPE degeneration.

Melatonin is known not only for its role in regulating human physiological functions (Muraleva et al., 2019), but also for its capacity to combat cellular oxidative stress (Reiter et al., 2016). A historical cohort analysis revealed that melatonin supplementation is linked to a decreased likelihood of developing and advancing AMD (Jeong et al., 2024). Kai Wang and colleagues have reported that melatonin mitigates NaIO3-triggered mitochondrial autophagy in ARPE-19 cells through the suppression of ROS-induced HIF-1α interaction with the BNIP3/LC3B signaling pathway (Wang et al., 2022). Nevertheless, the potential of melatonin to mitigate Aβ1-40-induced AMD through the inhibition of ferroptosis has yet to be elucidated. Our study explored the impact of melatonin on ferroptosis triggered by Aβ1-40 in the context of age-related macular degeneration. We found that melatonin significantly improved cell viability, reduced Aβ1-40-induced ferroptosis in RPE cells, and improved mitochondrial membrane density and size within the cells, as confirmed by transmission electron microscopy (Figure 3). Additionally, melatonin decreased the expression of intracellular Fe2+, ROS, and MDA, a lipid ROS byproduct, and reversed the abnormal expression of key ferroptosis biomarkers such as TFR1, ACSL4, SLC7A11, GPX4, and FTH1. These confirm that melatonin can attenuate Aβ1-40-induced degeneration of RPE cells by inhibiting ferroptosis *in vitro*.

Expanding our comprehension of the therapeutic potential of melatonin in AMD, we conducted *in vivo* studies to assess its effects on ferroptosis. Administering melatonin via intraperitoneal injections was found to safeguard photoreceptor cells against ferroptosis in a dry AMD model by curbing sodium iodate (NaIO3)-induced cell mortality (Zhi et al., 2024). In our *in vivo* experiments, we confirmed that melatonin ameliorated the disruption of the outer retinal structure and abnormalities in the RPE layer following intravitreal injection of Aβ1-40, and played a crucial role in preserving the integrity of the retinal structure (Figure 6). The primary goal of mitigating RPE cell damage is to protect retinal function and visual function. By conducting full-field ERG to assess changes in retinal function, we found that melatonin reversed the decline in a-wave and b-wave amplitudes induced by Aβ1-40 and demonstrated an improvement in retinal function. Additionally, we also observed that melatonin reversed the expression of ferroptosis-related factors such as TFR1, ACSL4, SLC7A11, and GPX4 at mRNA and protein expression levels. Our *in vitro* results indicated that melatonin significantly inhibited ferroptosis in AMD induced by Aβ1-40.

Our research delves deeper into the mechanisms by which melatonin mitigates ferroptosis associated with AMD. Lei Tang and colleagues have reported that melatonin deactivates microglial cells and enhances the stability of the inner blood-retinal barrier (iBRB) by suppressing the PI3K/Akt/Stat3/NF-κB signaling cascade (Tang et al., 2022). Our computational analysis further disclosed that the potential melatonin targets in AMD were predominantly associated with the KEGG pathways, particularly within the PI3K/AKT/MDM2 signaling cascade. Molecular docking experiments demonstrated that melatonin could form stable hydrogen bonds with PI3K, and its binding energy indicated a good interaction force (Figure 4). Fan Zhang et al. confirmed that overexpression of p53 could inhibit the expression of Slc7a11 and induce ferroptosis in the retina, while melatonin provided neuroprotective effects against retinal ischemia-reperfusion (RIR) injury by inhibiting p53-driven ferroptosis (Zhang F. et al., 2023). Ganxiao Chen et al. found that licorice chalcone A could alleviate ferroptosis in doxorubicin-induced cardiotoxicity through the PI3K/AKT/MDM2/p53 pathway (Chen et al., 2024). Therefore, we infer that melatonin can also inhibit ferroptosis and alleviate AMD through the PI3K/AKT/MDM2/P53 axis. Melatonin primarily influences ocular functions through its interaction with G protein-coupled receptors, specifically the melatonin receptor subtypes 1 (MT1) and/or 2 (MT2) (Felder-Schmittbuhl et al., 2024; Rosen et al., 2012). Both of these receptors are extensively distributed in the photoreceptors and retinal pigment epithelium of vertebrates, with their stimulation triggering distinct signaling cascades in a tissue-specific manner (Mehrzadi et al., 2020; Felder-Schmittbuhl et al., 2024). When melatonin concentrations are high, it can directly exert antioxidant effects and indirectly activate MT receptors in a combined manner to exert its effects (Rosen et al., 2012). At present, it is unclear if the cytoprotective action of melatonin on RPE cells is mediated through receptor-dependent or independent pathways, and additional studies are planned to elucidate this in the forthcoming period. Our research results indicate that melatonin can also reverse the expression of downstream molecules through the PI3K/AKT/MDM2/P53 axis, and low expression of P53 induces upregulation of Slc7a11, thereby inhibiting Aβ1-40-induced ferroptosis in RPE cells. Furthermore, when we knocked down the expression of MDM2, melatonin could no longer reverse P53-mediated ferroptosis. Subsequently, we observed similar manifestations by detecting lipid reactive oxygen species (ROS) (Figure 5). This confirms that melatonin inhibits ferroptosis in RPE cells through the PI3K/AKT/MDM2/P53 axis, further alleviating the occurrence of AMD.

In this study, we verified that Amyloid beta, one of the main components of drusen, can exacerbate RPE cell damage through ferroptosis, thereby accelerating the progression of AMD. Melatonin protects against Amyloid beta-induced RPE degeneration by inhibiting ferroptosis, and its mechanism of action is mediated through the PI3K/AKT/MDM2/P53 pathway. Previous research has demonstrated an association between melatonin use and a reduced risk of the onset and progression of Age-Related Macular Degeneration (AMD) (Jeong et al., 2024). Studies have suggested that daily administration of 3 mg of melatonin may protect the retina and delay macular degeneration (Yi et al., 2005), although the underlying mechanisms of its action remain incompletely understood. Our study indicates that targeting the specific death of Retinal Pigment Epithelium (RPE) cells may represent a novel approach for the prevention and treatment of AMD. Furthermore, we delve into the potential mechanisms by which melatonin acts as a candidate therapeutic agent in AMD treatment. There are also limitations in our study, such as whether the protective effect of melatonin on RPE cells involves receptor-dependent or receptor-independent mechanisms, which we will further investigate in future work.

## Data Availability

The data presented in the study are deposited in the ScienceDB repository, available at: https://www.scidb.cn/s/n22QJj.
